# Supplementary material for: Resolving the full spectrum of human genome variation using Linked-Reads
Source: Genome Res. 2019 Apr;29(4):635–45. doi: 10.1101/gr.234443.118 (PMC6442396; doi:10.1101/gr.234443.118)
Supplement: Supplemental Material [file supp_gr.234443.118_Supplemental_longranger-2.2.zip › longranger-2.2.tar.bz2!/lr2.2-public/lib/stan/cmdstan/stan/doc/api/html/classstan_1_1model_1_1prob__grad.html]

Stan: stan::model::prob\_grad Class Reference


|  |  |
| --- | --- |
|  | Stan  2.5.0  probability, sampling & optimization |


- Main Page
- Related Pages
- Namespaces
- Classes
- Files

- Class List
- Class Index
- Class Hierarchy
- Class Members

All Classes Namespaces Files Functions Variables Typedefs Enumerations Enumerator Friends Macros Pages

- stan
- model
- prob\_grad

Public Member Functions |
Protected Attributes |
List of all members

stan::model::prob\_grad Class Reference

The `prob_grad` class represents the basic parameter holders for a model.
More...

`#include <prob_grad.hpp>`

|  |  |
| --- | --- |
| Public Member Functions | |
|  | prob\_grad (size\_t num\_params\_r) |
|  | |
|  | prob\_grad (size\_t num\_params\_r, std::vector< std::pair< int, int > > &param\_ranges\_i) |
|  | |
| virtual | ~prob\_grad () |
|  | |
| size\_t | num\_params\_r () const |
|  | |
| size\_t | num\_params\_i () const |
|  | |
| std::pair< int, int > | param\_range\_i (size\_t idx) const |
|  | |

|  |  |
| --- | --- |
| Protected Attributes | |
| size\_t | num\_params\_r\_\_ |
|  | |
| std::vector< std::pair< int,   int > > | param\_ranges\_i\_\_ |
|  | |

## Detailed Description

The `prob_grad` class represents the basic parameter holders for a model.

The command `bin/stanc` builds Models extending this base helper class.

Definition at line 18 of file prob\_grad.hpp.

## Constructor & Destructor Documentation

|  |  |  |  |  |  |
| --- | --- | --- | --- | --- | --- |
| stan::model::prob\_grad::prob\_grad | ( | size\_t | *num\_params\_r* | ) |  |

inline

Definition at line 27 of file prob\_grad.hpp.

|  |  |  |  |
| --- | --- | --- | --- |
| stan::model::prob\_grad::prob\_grad | ( | size\_t | *num\_params\_r*, |
|  |  | std::vector< std::pair< int, int > > & | *param\_ranges\_i* |
|  | ) |  |  |

inline

Definition at line 32 of file prob\_grad.hpp.

|  |  |  |  |  |
| --- | --- | --- | --- | --- |
| virtual stan::model::prob\_grad::~prob\_grad | ( |  | ) |  |

inlinevirtual

Definition at line 38 of file prob\_grad.hpp.

## Member Function Documentation

|  |  |  |  |  |
| --- | --- | --- | --- | --- |
| size\_t stan::model::prob\_grad::num\_params\_i | ( |  | ) | const |

inline

Definition at line 44 of file prob\_grad.hpp.

|  |  |  |  |  |
| --- | --- | --- | --- | --- |
| size\_t stan::model::prob\_grad::num\_params\_r | ( |  | ) | const |

inline

Definition at line 40 of file prob\_grad.hpp.

|  |  |  |  |  |  |
| --- | --- | --- | --- | --- | --- |
| std::pair<int,int> stan::model::prob\_grad::param\_range\_i | ( | size\_t | *idx* | ) | const |

inline

Definition at line 48 of file prob\_grad.hpp.

## Member Data Documentation

size\_t stan::model::prob\_grad::num\_params\_r\_\_

protected

Definition at line 22 of file prob\_grad.hpp.

std::vector<std::pair<int,int> > stan::model::prob\_grad::param\_ranges\_i\_\_

protected

Definition at line 23 of file prob\_grad.hpp.

---

The documentation for this class was generated from the following file:

- src/stan/model/prob\_grad.hpp


---

[ Stan Home Page ]

*© 2011–2014,
Stan Development Team.*
